# Health economic evaluation of vaccination strategies for the prevention of herpes zoster and postherpetic neuralgia in Germany

**DOI:** 10.1186/1472-6963-13-359

**Published:** 2013-09-26

**Authors:** Bernhard Ultsch, Felix Weidemann, Thomas Reinhold, Anette Siedler, Gérard Krause, Ole Wichmann

**Affiliations:** 1 Immunisation Unit, Robert Koch Institute, Berlin, Germany; 2 Charité University Medical Centre, Berlin, Germany; 3 Institute for Social Medicine, Epidemiology and Health Economics, Charité - University Medical Centre, Berlin, Germany; 4 Department for Infectious Disease Epidemiology, Robert Koch Institute, Berlin, Germany; 5 Department for Epidemiology, Helmholtz Centre for Infection Research, Brunswick, Germany

**Keywords:** Herpes zoster, Postherpetic neuralgia, Vaccine, Cost-effectiveness, QALY, Markov

## Abstract

**Background:**

Herpes zoster (HZ) is a self-limiting painful skin rash affecting mostly individuals from 50 years of age. The main complication is postherpetic neuralgia (PHN), a long-lasting pain after rash has resolved. A HZ-vaccine has recently been licensed in Europe for individuals older than 50 years. To support an informed decision-making for a potential vaccination recommendation, we conducted a health economic evaluation to identify the most cost-effective vaccination strategy.

**Methods:**

We developed a static Markov-cohort model, which compared a vaccine-scenario with no vaccination. The cohort entering the model was 50 years of age, vaccinated at age 60, and stayed over life-time in the model. Transition probabilities were based on HZ/PHN-epidemiology and demographic data from Germany, as well as vaccine efficacy (VE) data from clinical trials. Costs for vaccination and HZ/PHN-treatment (in Euros; 2010), as well as outcomes were discounted equally with 3% p.a. We accounted results from both, payer and societal perspective. We calculated benefit-cost-ratio (BCR), number-needed-to-vaccinate (NNV), and incremental cost-effectiveness ratios (ICERs) for costs per HZ-case avoided, per PHN-case avoided, and per quality-adjusted life-year (QALY) gained. Different target age-groups were compared to identify the most cost-effective vaccination strategy. Base-case-analysis as well as structural, descriptive-, and probabilistic-sensitivity-analyses (DSA, PSA) were performed.

**Results:**

When vaccinating 20% of a cohort of 1 million 50 year old individuals at the age of 60 years, approximately 20,000 HZ-cases will be avoided over life-time. The NNV to avoid one HZ (PHN)-case was 10 (144). However, with a BCR of 0.34 this vaccination-strategy did not save costs. The base-case-analysis yielded an ICER of 1,419 (20,809) Euros per avoided HZ (PHN)-case and 28,146 Euros per QALY gained. Vaccination at the age of 60 was identified in most (sensitivity) analyses to be the most cost-effective vaccination strategy. In DSA, vaccine price and VE were shown to be the most critical input-data.

**Conclusions:**

According to our evaluation, HZ-vaccination is expected to avoid HZ/PHN-cases and gain QALYs to higher costs. However, the vaccine price had the highest impact on the ICERs. Among different scenarios, targeting individuals aged 60 years seems to represent the most cost-effective vaccination-strategy.

## Background

Before implementation of routine childhood varicella vaccination in 2004, the lifetime risk of acquiring a varicella-zoster-virus (VZV) infection in Germany was almost 100% [1,2]. Children under 10 years of age were predominantly affected by the infection, which clinically manifests as chickenpox (varicella). After recovery from varicella, the virus remains latent life-long in individual’s dorsal root ganglia [3]. Later in life, the VZV can reactivate and manifest as shingles (herpes zoster, HZ) as a result of decreasing VZV-specific T-cell-immunity [4,5]. Natural waning immunity and other causes like psychological stress or immunosuppression can contribute to a VZV-reactivation, too [6].

HZ is a painful and self-limiting skin-rash that lasts approximately four weeks [7-9]. The main complication of HZ is postherpetic neuralgia (PHN) a long-lasting pain in the formerly HZ-affected skin region after rash has resolved [5,10-12]. Both, HZ and PHN cause limitations on quality of life (QoL) [13-16]. Approximately 20-30% of individuals experience at least one HZ-episode in their life [17]. The risk and burden of HZ increase with age, most cases being 50 years and older [4,18-21]. In Germany, the risk in this age-group is estimated at 9 per 1,000 persons per year (PY), which is comparable to other industrialized countries [21-23]. Estimates for the proportion of HZ-patients developing PHN range between 6 and 7% [21,23]. The options for therapy and prevention of HZ and PHN are limited [24-28].

In 2006, the first vaccine for the prevention of HZ and PHN for people 50 years of age and older was licensed in Europe [12]. This live-attenuated vaccine demonstrated its efficacy in two double-blinded, placebo-controlled randomized trials (RCT), which included healthy individuals aged >50 years [29,30]. Another HZ-vaccine candidate is currently tested in clinical phase III trials and might be available in the near future [31]. As of June 2013, the licensed vaccine was not yet available on the market in most European countries. However, the market launch in Germany might be expected soon. To support an informed decision-making of a potential vaccine-recommendation by relevant authorities, we performed a health economic evaluation of routine HZ-vaccination among the elderly and compared various vaccination strategies.

## Methods

### Model design

**Figure 1:**
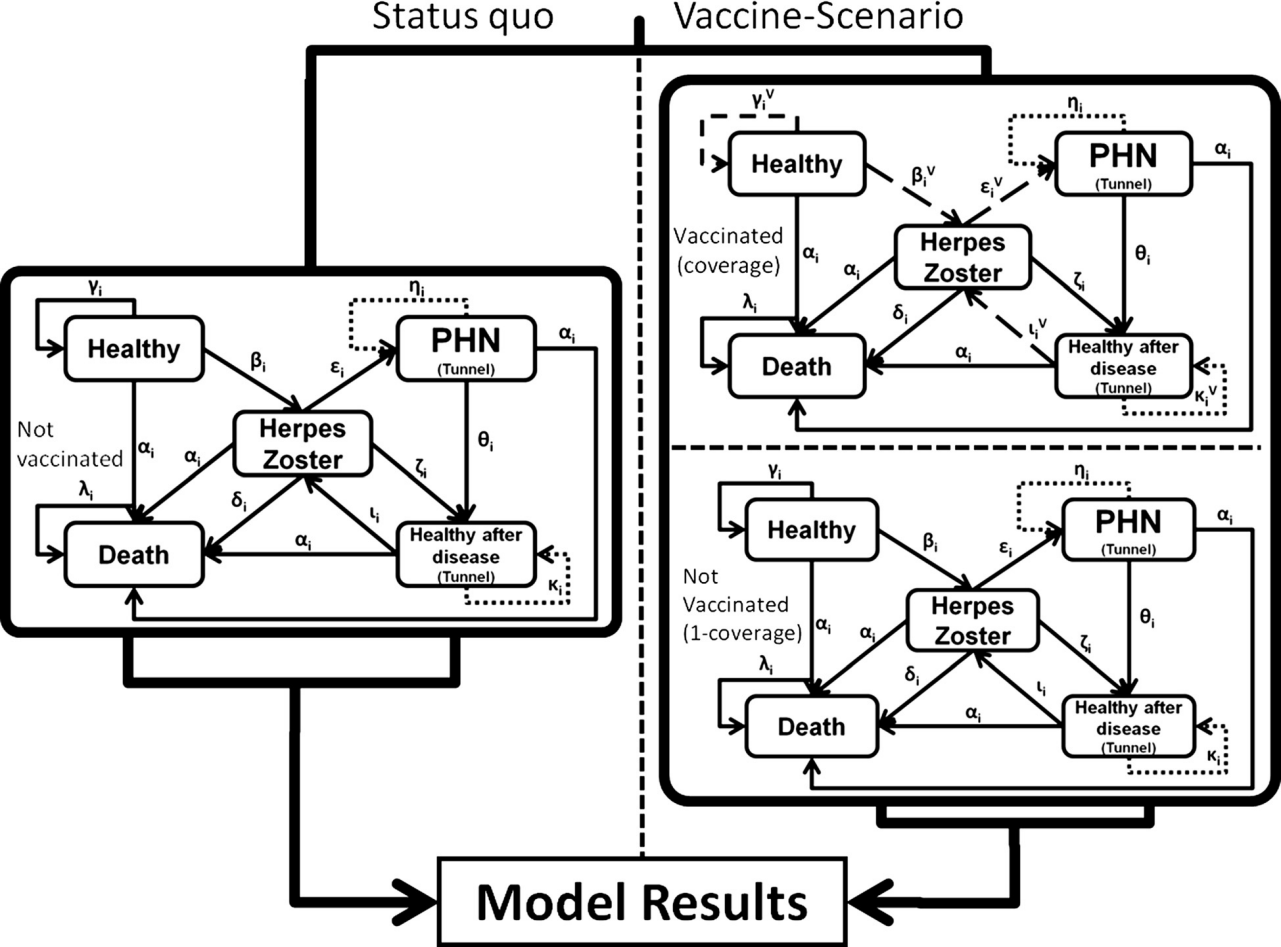
Markov-model structure.

We developed a static Markov-cohort model, since no transmission dynamics like herd protection after implementation of HZ-vaccination can be expected [32]. In the model, a scenario with routine HZ-vaccination in the German statutory health insurance (SHI) system (‘vaccine-scenario’) was compared with the current situation without HZ-vaccination (‘status quo’). The SHI system covers approximately 85% (70 million individuals) of the German living population [33]. According to the disease course, we modeled five Markov-states: ‘Healthy’, ‘Herpes zoster’, ‘PHN’, ‘Healthy after Disease’, and ‘Death’ (Figure 1). States were connected by transition probabilities (confer arrows and respective Greek letters shown in Figure 1 and Table 1). The state ‘Death’ was an absorbing state and ‘PHN’ as well as ‘Healthy after Disease’ were ‘tunnel’-states such that individuals stayed a fixed time in these states. We defined the Markov-cycle length to be three months, since HZ usually lasts about one month and a potential PHN occurs proximately three months after rash onset and lasts nine months by average [7-9,12,13,34-41]. The model started with a cohort of 1 million individuals at 50 years of age with a life-long time-horizon. For each scenario we calculated the number of HZ-cases and PHN-cases, as well as quality-adjusted life-years (QALYs). Based on the accounted costs we calculated the benefit-cost ratio (BCR) comparing the two scenarios. In addition, we calculated the number-needed-to-vaccinate (NNV) and incremental cost-effectiveness ratios (ICERs) accounting for costs per HZ- and PHN-case avoided and per QALY gained. We modeled the payer perspective (PP), which includes only direct costs from the view of the SHI as well as the societal perspective (SP), which includes direct plus indirect costs: sick leave and patient’s co-payments [42,43].

**Table 1:** Transition probabilities

**State (From state** → **To state)**	**Variable**	**Formula**
**Death**		
Death → Death	λ_i_	=1
Any state → Death	α_i_	= $p_{i}^{\text{Background}\;\text{Mortality}}$
	**Status quo [no vaccination]**	
**Healthy**		
Healthy → Herpes zoster	β_i_	= $\left(1-\alpha_{i}\right)*\left(1-e^{-I_{i}^{\text{Healthy}\to \text{HZ}}}\right)$
Healthy → Healthy	γ_i_	= 1 - *α*_i_ - *β*_i_
**Herpes zoster**		
Herpes Zoster → Death	δ_i_	= $\left(1-\alpha_{i}\right)*\left(1-e^{-I_{i}^{\text{HZ}\to \text{Death}}}\right)$
Herpes Zoster → PHN	ϵ_i_	= $\left(1-\alpha_{i}\right)*\left(1-e^{-I_{i}^{\text{HZ}\to \text{PHN}}}\right)$
Herpes Zoster → Healthy after disease	ζ_i_	= 1 - *α*_i_ - *δ*_i_ - ϵ_i_
**PHN**		
PHN → PHN	η_i_	= $\chi_{\left[1,D^{\text{PHN}}\right]\;}\left(\sigma \right)*\left(1-\alpha_{i}\right)$
PHN → Healthy after disease	θ_i_	= 1 - *α*_i_ - η_i_
**Healthy after disease**		
Healthy after disease → Healthy after disease	κ_i_	= $\chi_{\left[1,D^{\text{Rec}}\right)}\;\left(\sigma \right)*\left(1-\alpha_{i}\right)+\chi_{\left[D^{\text{Rec}},\infty \right)}\;\left(\sigma \right)*\gamma_{i}$
Healthy after disease → Herpes zoster	ι_i_	= 1 - *α*_i_ - *κ*_i_
	**Vaccine-Scenario [adapted transition probability due to vaccine efficacy (VE)]**	
**Healthy**		
Healthy → Herpes zoster	β_i_^V^	= $\beta_{i}*\left(1-{\mathrm{VE}}_{i}^{\mathrm{HZ}}\right)$
	VE_i_^HZ^	= $\chi_{\left[j,\mu \right)}\;\left(i\right)*{\mathrm{IVE}}_{j}^{\mathrm{HZ}}+\chi_{\left[\mu ,\infty \right)}\;\left(i\right)*{\mathrm{IVE}}_{j}^{\mathrm{HZ}}*e^{-\pi *\left(i-\mu \right)}$
Healthy → Healthy	γ_i_^V^	= $\;1-\alpha_{i}-\beta_{i}^{V}$
**HZ**		
Herpes zoster → PHN	ϵ_i_^V^	= $\epsilon_{i}*\left(1-{\mathrm{VE}}_{i}^{\mathrm{PHN}}\right)$
	VE_i_^PHN^	= $\chi_{\left[j,\mu \right)}\;\left(i\right)*{\mathrm{IVE}}_{j}^{\mathrm{PHN}}+\chi_{\left[\mu ,\infty \right)}\;\left(i\right)*{\mathrm{IVE}}_{j}^{\mathrm{PHN}}*e^{-\pi *\left(i-\mu \right)}$
**Healthy after disease**		
Healthy after Disease → Healthy after disease	κ_i_^V^	= $\chi_{\left[1,D^{\mathrm{Rec}}\right)}\;\left(\sigma \right)*\left(1-\alpha_{i}\right)+\chi_{\left[D^{\mathrm{Rec}},\infty \right)}\;\left(\sigma \right)*\gamma_{i}^{V}$
Healthy after Disease → Herpes zoster	ι_i_^V^	= $1-\alpha_{i}-\kappa_{i}^{V}$

*p* = probability of death with respect to cycle-length; i = age = Start_Age +(n_cycles); *χ*_A_(x) = Indicator function; σ = Tunnel (counter for cycles staying in state); D^PHN^ = Duration of PHN; D^Rec^ = Duration until Recurrence is possible; v = indicating the vaccine scenario; j = Age at Vaccination; μ = Age at Vaccination plus n years; I = Age-group specific Incidence^from_state→to_state^ with respect to cycle-length; VE_i_^HZ^ = Vaccine efficacy protecting against HZ; IVE_j_^HZ^ = Initial Vaccine efficacy protecting against HZ by age at vaccination; VE_i_^PHN^ = Vaccine efficacy protecting against PHN by certain age in model; IVE_j_^PHN^ = Initial Vaccine efficacy protecting against PHN by age at vaccination; π = Waning rate with respect to cycle-length.

### Input-data

#### Epidemiology

When considering a new vaccine in health economic evaluations, the epidemiology of the target disease is one of the most critical input factors. For our model, HZ- and PHN-related incidence data specific for the German context were derived from a recent retrospective data analysis in Germany [21]. The anonymized data set of AOK Hesse (SHI funds) contained about 240,000 insured individuals including their diagnoses- and service utilization-data for the year 2004 to 2009. Acute outpatient HZ-cases were identified via ‘International Statistical Classification of Diseases’ (ICD-10) B02.*/G53.0 with an additional diagnosis reliability ‘assured’ or ‘conjectured’. Inpatient HZ-cases were identified via German diagnoses-related groups (G-DRG). PHN was in accordance to other studies defined as pain persisting at least three month after HZ-onset [12,29,35-41]. Besides respective ICD-10 codes, HZ-cases that developed PHN were identified via PHN-specific pain medication based on German guidelines [28]. About 5.79 HZ-cases per 1,000 person-years (PY) were observed annually. Of these HZ-cases about 5 % developed PHN [21]. Exact HZ-incidence and PHN-proportion figures were provided in Table 2.

**Table 2:** Model input-data

	**Base-case**				**DSA**				**PSA**
	**HZ**		**PHN**		**HZ**		**PHN**		
**Epidemiology**[21]									
**Age-group**	Cases/1,000 PY		% of HZ-cases		95% CI (Cases/1,000 PY)		95% CI (% of HZ-cases)		Triangular
**50–59**	6.56		3.38		6.12–7.01		2.15–4.61		
**60–69**	9.19		4.92		8.68–9.71		3.73–6.11		
**70–79**	11.24		7.85		11.66–11.82		6.48–9.23		
**>80**	12.76		7.8		11.92–13.61		6.05–9.55		
**Vaccine efficacy (%)**[29,30,50,51]									
**Age-group**					95% CI		95% CI		Beta
**50–59**	69.8		65.7*		54.1–80.6		20.4*–86.7*		
**60–64**	65.4		65.7		55–70.9		20.4–86.7		
**65–69**	62.58		65.7		55–70.9		20.4–86.7		
**70–74**	43.74		66.8		25–48.1		43.3–81.3		
**75–79**	36.51		66.8		25–48.1		43.3–81.3		
**80–84**	20.09		66.8		25–48.1		43.3–81.3		
**>85**	13.22		66.8		25–48.1		43.3–81.3		
**EQ-5D utilitiesª**[14]									
**Age-group**					95% CI		95% CI		Beta
**50–60**	0.68		0.725		0.61–0.745		0.63–0.815		
**61–70**	0.6		0.68		0.525–0.67		0.605–0.76		
**>70**	0.62		0.64		0.550.69		0.57–0.705		
**Treatment costs per case (€)**[21]									
**Perspectives/Age-group**	Payer	Societal	Payer	Societal	Payer 95% CI	Societal 95% CI	Payer 95% CI	Societal 95% CI	Gamma
**50–59**	193	570	872	1,339	148–245	495–651	289–1,940	436–3,065	
**60–69**	226	338	1,349	2,137	179–270	281–397	714–2,125	975–3,625	
**70–79**	203	214	1,172	1,218	159–252	169–263	717–1,785	750–1,863	
**>80**	320	331	642	676	249–394	259–408	251–1,157	270–1,207	
	Background mortality [probability (%) p.a.]***[48]					HZ related mortality [deaths/100,000 PY]***[22]			
**Age-group**									
**50–59**			0.7				0.02		
**60–69**			1.5				0.09		
**70–79**			3.9				0.42		
**80–89**			11				2.53		
**90–99**			26.2				3.86		
**100+**			56.8				3.86		
**Other input parameter**									
	**Base-case**				**DSA [lower/higher value]**				**PSA**
**Vaccine Price/dose (€)**[59]	140.48				70.24		210.72		Normal
Vaccine administration costs (€)**	7				5.20		10.81		Normal
**Discount rate (% p.a.)**	3				0		10		
**Waning immunity rate (% p.a.)**[56]	8.3				0		20		Beta
**Coverage (%) assumption**	20				-		-		
HZ susceptibility**** (%)[2]	100				-		-		
**EQ-5D utilities for healthy individuals (age in years)**	1(all ages)				0.859^b^ (41–60)		0.7684^b^ (61+)		

*DSA* Deterministic Sensitivity Analysis, *PSA* Probabilistic Sensitivity Analysis displays the age related probability distribution of respective input-data used in Monte Carlo simulations, *CI* confidence interval, *value adopted from age 60; **vaccine administration costs adopted from pneumococcal vaccination (adjusted from different regional values); ***Values adjusted to table’s age-groups; ****All individuals in the model were susceptible for developing HZ due to high seroprevalence of varicella-zoster virus in the German population; ªAdjusted from Drolet et al. [14]; ^b^Gender unspecific values adjusted from linear model in Hinz et al. [61]; *€*, Euro in 2010 price levels; *PY* persons per year.

Several studies have assessed the risk of HZ-recurrence, i.e. the occurrence of a subsequent HZ-episode [4,20,44-47]. There are discrepant findings on the frequency of this phenomenon in the literature. However, studies suggesting that the risk of HZ-recurrence is low have a rather short follow-up time and/or a low number of HZ-patients included [20,44,46]. In contrast, studies with a follow-up period of several years conclude consistently that the risk of a subsequent HZ-episode is a critical factor when considering HZ-related disease burden [4,45,47]. A large study addressing specifically the risk of HZ-recurrence, which involved 1,669 HZ-cases with an average follow-up of 7.3 years, suggests that the incidence of HZ-recurrence is similar to the incidence of a first HZ-episode [47]. We have accounted for this risk by using same input-data for HZ-incidence and HZ-recurrence (see Sections ‘Base-case analysis’ and ‘Structural sensitivity analysis’ for further exemplifications). Incidence of HZ-associated death was available from a retrospective data-analysis in Germany [22]. The probability of death due to all causes by age (‘background mortality’) in the general population living in Germany was derived from public statistics of the federal statistical office in Germany [48] (Table 2).

#### Utility data

For cost-utility analyses, quality of life (QoL) data were considered. Since HZ-related QoL and pain intensity data among HZ- or PHN-patients is lacking for Germany, we used data from a study by Drolet and colleagues, which was conducted in a Canadian population of individuals 50 years of age and older without stratification by HZ severity [14]. This fitted well to our input-data (incidence and costs), which were derived from all patients without specification of pain intensity. We informed our model with the corresponding ‘EQ-5D scores’ (utilities). We fitted the data by calculating the age-specific average utilities based on values of day 0 and day 30 as HZ-related utilities, and the age-specific average based on values of day 90 and day 180 as PHN-related utilities, as defined by Drolet et al. [14]. For individuals located in the states ‘Healthy’ and ‘healthy after disease’ we chose 1 as a value for baseline utility in the base-case. To account for vaccine-related adverse reactions on the QoL, we assumed a utility of 0.99 for two days per vaccinee, since the vaccine seems to be generally well tolerated [29,49].

#### Vaccine characteristics

**Figure 2:**
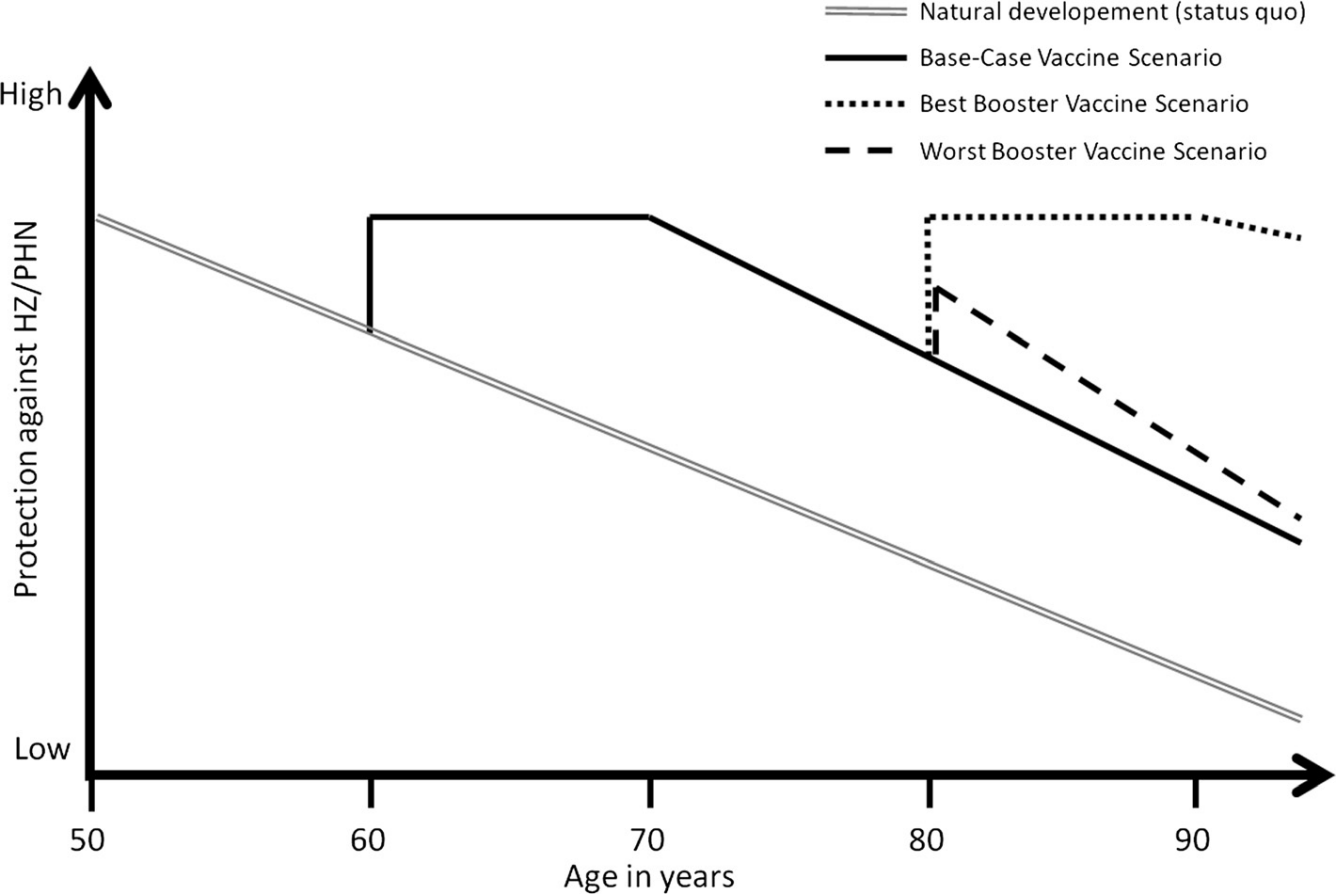
**Schematic presentation of waning-immunity after VZV-infection and after additional vaccination with Herpes-zoster vaccines with/without booster-scenarios.** HZ, herpes zoster; PHN, postherpetic neuralgia.

Vaccine efficacy (VE) as another key factor in this health economic analysis was utilized carefully. Published data from the currently licensed live-attenuated vaccine were considered for VE: data for preventing HZ and PHN in elderly was derived from the RCT ‘shingles prevention study’ (SPS) involving almost 40,000 individuals 60 years of age and older [29]. In this study, the average VE in preventing HZ was 51.3% and in preventing PHN 66.5% [29,50]. For HZ-related VE stratified by more precise age-groups, we considered information from the clinical briefing document of the US Food and Drug Administration (FDA) [51] as well as a recent RCT in individuals aged 50–59, where HZ-related VE was 69.8% [30]. As this RCT did not measure the PHN-related VE, we adopted this value from the age-group 60–69 years from SPS (Table 2). A sub-study of the SPS re-enrolled over 14,000 subjects (7,320 vaccine and 6,950 placebo recipients) in the short-term persistence sub-study (STPS) in order to assess the persistence of VE over time since vaccination [52,53]. VE for HZ and PHN was assessed for the combined SPS and STPS populations as well as for each year through year 7 after vaccination. The overall HZ (PHN)-VE in the SPS+STPS population was 48.7% (64.9%). However, during the 7 year follow-up, a certain decline in HZ-related VE and a variation in VE concerning PHN were observed. HZ-related VE ranged through years between 62 and 30.6%; PHN-related VE alternated between 83.4 and 32% [53]. Yet, these results were not statistically significant since confidence intervals overlapped. Thus, we assumed a stable VE for 10 years following vaccination. However, to use conservative assumptions, we considered in our model a ‘waning’ of vaccine induced immunity as done elsewhere [40,54,55]. After 10 years of stable VE, HZ- and PHN-related VE decreased exponentially by 8.3% per year (Table 1, Table 2, and Figure 2) [56]. Coverage was assumed to be 20% since pneumococcal vaccination coverage among elderly in Germany is with 10-29% relatively low, and since the HZ-vaccine coverage in the US was 15.8% in 2011 [57,58].

#### Cost data

Cost data was derived from the same study where the incidence data was utilized. Data was stratified by HZ and PHN, by age-groups, and by perspectives (Table 2). As all cost values in our model were at Euro 2010 price levels, we considered the German pharmacy retail price of HZ-vaccine for the same year reduced by the obligatory pharmacy and manufacturer discounts. Thus, the HZ-vaccine price per dose in our model was set to 140.48 Euros [59]. We used the mean administration costs per pneumococcal vaccine dose in Germany in 2010 (7 Euros) as a proxy for HZ-vaccine administration in the base-case assumption and considered the regional minimum and maximum values for sensitivity analyses (SA, Table 2).

All epidemiological, demographic, and VE data were, if necessary, converted into age-group specific probabilities or relative risks with respect to the model’s cycle-length (see Table 1 and Figure 1) [60].

### Base-case analysis

In base-case analysis, the model’s start age was set to 50 years and the age of vaccination to 60 years. For incidence and recurrence rates as well as for treatment costs per case we used the respective point estimators from studies as presented in ‘Input data’ section and Table 2. For the tunnel state ‘Healthy after Disease’, we defined that a recurrence cannot occur within three years (12 cycles) after preceding the first HZ-episode [47]. Furthermore we adjusted the tunnel state ‘PHN’ to keep individuals three cycles, since the average duration of PHN is 9 month (confer ‘Model design’ section). The utility values, waning rate, vaccine price, and vaccine honorary were set as described in ‘Input data’ section. For ICER calculation, costs and outcomes were discounted equally with 3% per annum as suggested in Germany [42] (Table 2). Additionally we conducted a ‘Break-even’ analysis. For this we used the base-case conditions to identify the vaccine price at which the ICERs represent cost-savings.

### Univariate deterministic sensitivity analyses

With univariate deterministic sensitivity analyses (DSA) we investigated the impact of different age-specific incidences and treatment costs, VE, waning immunity rates, discount rates, and vaccine prices as well as administration costs and utility values (Table 2). For measuring the impact of an absent HZ-recurrence we set the respective value to zero. To measure the impact of a high HZ-recurrence, we used the upper limit 95% confidence interval (CI) of HZ-incidence values and removed the ‘tunnel’-state condition. Hence, in the latter scenario a HZ-episode was theoretically possible right after the previous one. Since in a static model a change in coverage would not affect the ICERs, we neglected this variation in the SA. In contrast to the base-case we changed the age of vaccination from 60 years to 50, 55, 65, 70, 75, and 80 years and stratified the analyses by outcome and perspective.

### Structural sensitivity analysis

In a *‘Best Case Scenario’* we assumed a situation in favor of the new HZ-vaccine and changed input-data accordingly. We used the higher values of 95% CI of the HZ and PHN disease-incidences and costs per case. Furthermore, we set the recurrence rate as high as described before and used the lower utilities for HZ and PHN, respectively. Higher 95% CI values were taken for VE whereas the waning rate was set to zero. Vaccine administration costs were lowered to 5.20 Euros and the vaccine price by 50%.

The *‘Worst case Scenario’* represented possible least preferable results. In terms of epidemiology we used the lower 95% CI estimates of the HZ- and PHN-incidence. For HZ and PHN-treatment costs we took the lower 95% CI values and neglected recurrence by setting its probability to zero. We considered higher utilities, an annual waning rate of 20% and the lower 95% CI values of the VE. We increased the vaccine price by 50% and set the administration costs to 10.81 Euros per vaccination.

The variation of *‘Duration of stable VE’* aims to reduce the potential uncertainty associated with the model’s base-case that considers 10 years of stable VE following vaccination. Since this is an assumption based on trial data (see section ‘Vaccine characteristics’) we conducted analyses where we changed the period of stable VE following vaccination from 0, 5, 15, to 20 years.

A combined analysis of the impact of different annual waning immunity rates (1%, 5%, 8.3%, and 20%), duration of stable VE (0, 5, 10, and 15 years), and the age at vaccination (50, 60, and 70 years) on the costs per QALY gained was performed to identify the optimal age at vaccination.

Furthermore, we implemented different ‘*booster scenarios’* into the model. Therefore, HZ-vaccinated individuals were administrated a second dose 20 years after first HZ-vaccination. This booster was given to 50% of these formerly HZ-vaccinated individuals located in the states ‘Healthy’ or ‘Healthy after disease’ within the vaccine-scenario (Figure 1). Besides additional costs due to vaccination and a two day reduction of individual’s QoL because of vaccine related adverse reactions the booster shot increased the vaccine-induced immunity. We modeled two oppositional booster scenarios: In favor for the HZ-vaccine and its booster we assumed in the *‘Best Case Booster Scenario’* an increase of the vaccine-induced immunity to the same level as after the first vaccination (Table 2), and a constant VE for 10 years before the waning with a rate of 1% p.a. began (Figure 2). In the *‘Worst Case Booster Scenario’* the booster increased the vaccine induced immunity only to the respective level of the actual age as presented in RCTs considering VE (Table 2) and this immunity diminished immediately by the annual waning rate of 20%. In order to account for the influence of age at vaccination on the ICERs, we modeled both booster scenarios not only for the base-case (60 years of age at vaccination) but also for 50, 70, and 80 years at vaccination.

To measure the impact of varying ‘*duration of PHN’* on ICER (cost per QALY gained), we changed PHN duration from 9 months (base-case) to 6, 15, and 36 month, respectively.

In order to assess the impact of a ‘*change in baseline utility’* values for healthy individuals, we used data from a study conducted in Germany [61]. In this study 2,022 healthy individuals between 16 and 93 years of age were interviewed using the EQ-5D questionnaire. Furthermore three different accounting models (sum model, linear model, and a multiplicative model) were used to calculated values resulting from EQ-5D items. For this SA we used the age-specific utilities from the linear model [61]. Since our model uses throughout gender unspecific input-data, we calculated age-specific average values from both gender (Table 2).

### Multivariate probabilistic sensitivity analyses

To identify the robustness of the base-case and the probability of extreme favorable or unfavorable results and to get more insights into uncertainties, we performed multivariate probabilistic sensitivity analyses (PSA) [62]. We conducted a Monte Carlo simulation with 10,000 runs for each outcome (HZ- and PHN-cases avoided, QALY gained). The respective input-data were drawn simultaneously (multivariate) from probability distributions within the ranges considered in DSA (Table 2). For guidance purposes four thresholds (20,000, 30,000, 50,000, and 100,000 Euros per QALY gained) representing different fictitious willingness to pay (WTP) levels were implemented within the scatter plot of the PSA considering costs per QALY gained.

### External comparison and software

For a proper external comparison of our model results we considered findings from other international health economic evaluations. For a better comparison, we inflated the cost data from other studies to price levels of 2010 (reference year) within each study’s country using consumer prices indices and converted these respective amounts by the gross domestic product purchasing power parity (GDP PPP) into Euro amounts with Germany as reference country based on data from the Organization for Economic Co-operation and Development (OECD) [63,64]. Hence, amounts were presented in PPP-Euros. Furthermore, we considered those analyses in respective studies that were similar to our base-case. Thus, we did not necessarily refer to base-case results from the other studies in the ‘Discussion’ section.

Software used was: TreeAge^®^ Pro 2012 (TreeAge Software, Williamstown, MA), Microsoft^®^ Excel 2010 (Microsoft, Redmond, WA), and STATA^®^ 12.1 (StataCorp, College Station, TX).

## Results

### Base-case results

**Figure 3:**
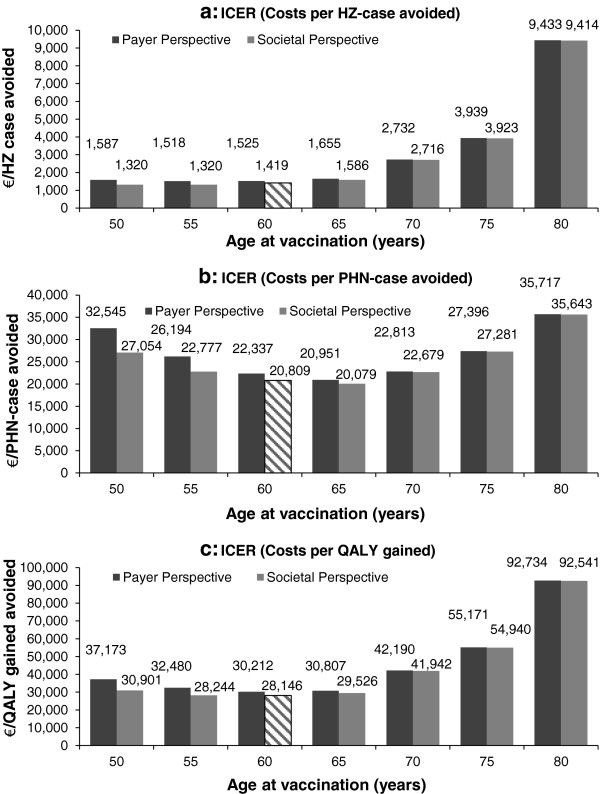
**Base-case-analyses and variation of age and perspective - ICERs by outcome and age.** ICER, incremental cost-effectiveness ratio; HZ, herpes zoster; PHN, postherpetic neuralgia; QALY, quality adjusted life-year. NOTE: Hatched bars represent base-case analyses.

Vaccinating 20% of individuals in a cohort of 1 million 50 year old persons at the age of 60 years costs almost 30 million Euros. Over the cohort’s lifetime approximately 20,000 HZ-cases can be avoided, which results into a reduction of treatment costs by over 10 million Euros. These figures translate in a BCR of 0.34, which indicates that HZ-vaccination is not a cost-saving measure (Table 3a). Ten individuals need to get vaccinated to avoid one HZ-case and 144 to avoid one PHN-case. In terms of gaining one QALY, 195 people need to receive a shot (Table 3b). As indicated by the BCR costs in the vaccine scenario are higher than in status quo. This is because besides costs for vaccination, costs for treatment (however, with a lower probability and therefore proportion) do occur in vaccination scenario, too. The QALY gain is also higher in the vaccination scenario (Table 3c). Considering the ICERs, the vaccination of 60 year old individuals yield costs of 1,419 Euros per HZ-case avoided, 20,809 Euros per PHN-case avoided, and 28,146 Euros per QALY gained (Figure 3 and Table 4). As indicated by the ‘Break-even’ analysis, a price per vaccine dose below 26.50 Euros (i.e. >80% below base-case assumption) would result in potential cost-savings.

**Table 3:** Results from base-case analyses

**a: Overall effects [cohort of 1 million individuals not discounted]**					
	**Number of HZ-cases**	**Costs of treatment (€)**	**Costs of vaccination (€)**	**Total costs (€)**	BCR*
**Status quo**	284,768	127,978,000	-	127,978,000	
**Vaccination scenario**	264,977	117,948,913	29,496,000	147,444,913	0.34
**b: Number needed to vaccinate (NNV)**					
**Avoiding one HZ-case**		10			
**Avoiding one PHN-case**		144			
**Gaining one QALY**		195			
**c: Costs and effects in both scenarios of the model**					
Not discounted		**Costs (€)**	**Effects [QALY]**	**Costs/Effects**	**ICER**
**Status quo**		127.98	29.2747	4.3716	
**Vaccination scenario**		147.44	29.2757	5.0364	19,002
Discounted					
**Status quo**		82.80	18.8569	4.3911	
**Vaccination scenario**		98.64	18.8574	5.2307	28,146

*Benefit-Cost ratio; *€*, Euro in 2010 price levels.ICER, incremental cost-effectiveness ratio; HZ, herpes zoster; PHN, postherpetic neuralgia; QALY, quality adjusted life-year.

**Table 4:** Results from structural sensitivity analyses (discounted)

**a: Extreme scenarios and variations in the duration of stable VE**					
	**Base-case**			**Extreme scenario**	
				**Best case**	**Worst case**
ICER by outcome					
**€/HZ-case avoided**	1,419			17	4,595
**€/PHN-case avoided**	20,809			163	327,691
**€/QALY gained**	28,146			235	157,845
**b: Duration of stable VE [years]**					
	**0**	**5**	**10 (base-case)**	**15**	**20**
ICER by outcome					
**€/HZ-case avoided**	2,633	1,800	1,419	1,225	1,123
**€/PHN-case avoided**	40,300	27,057	20,809	17,708	16,128
**€/QALY gained**	53,702	36,211	28,146	24,060	21,931
**c: Booster scenarios [by age at vaccination in years]**					
	**Base-case**	**Best case**			
		**50**	**60**	**70**	**80**
ICER by outcome					
**€/HZ-case avoided**	1,419	1,080	1,361	2,701	9,372
**€/PHN-case avoided**	20,809	19,001	19,632	22,931	35,604
**€/QALY gained**	28,146	30,241	32,173	46,126	93,497
	**Base-case**	**Worst case**			
		**50**	**60**	**70**	**80**
ICER by outcome					
**€/HZ-case avoided**	1,419	1,580	1,621	2,792	9,384
**€/PHN-case avoided**	20,809	29,095	22,643	23,345	35,619
**€/QALY gained**	28,146	52,628	39,604	47,559	93,565
**d: Variations in the length of PHN [month]**					
	**6**	**9 (base-case)**	**15**	**36**	
ICER by outcome					
**€/QALY gained**	31,741	28,146	23,048	4,149	

*€*, Euro in 2010 price level.ICER, incremental cost-effectiveness ratio; HZ, herpes zoster; PHN, postherpetic neuralgia; QALY, quality adjusted life-year; VE, vaccine efficacy.

### Univariate deterministic sensitivity analyses

Considering the age at vaccination, cost-effectiveness is decreasing (indicated by higher ICERs) between the age of 55 and 75 years. Due to low VE, vaccination of 80 year old individuals seems least cost-effective, especially when considering costs per HZ-case avoided (Figure 3a). In terms of PHN-cases avoided the most cost-effective age at vaccination is 65 years. However, the base-case results are only slightly higher (hatched bar in Figure 3b). The lowest ICER with respect to QALYs gained is represented by the base-case (hatched bar in Figure 3c). Due to indirect costs occurring in the working population, the difference between PP and SP is higher between 50 to 60 year old individuals and decreases with increasing age.

**Figure 4:**
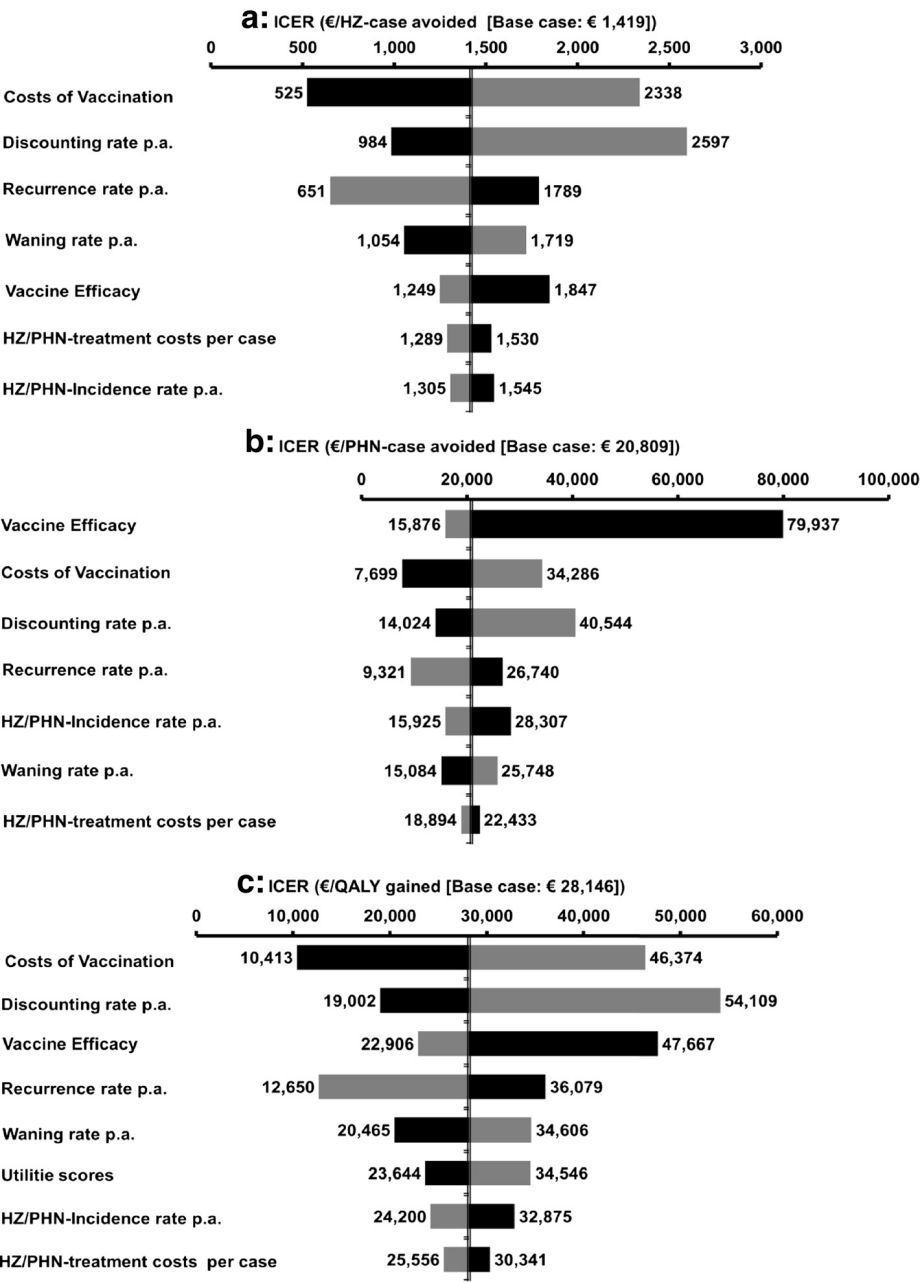
**Deterministic sensitivity analysis by outcomes from societal perspective.** ICER, incremental cost-effectiveness ratio; HZ, herpes zoster; PHN, postherpetic neuralgia; QALY, quality adjusted life-year; p.a., per annum. NOTE: Black bars represent lower input values.

The costs of vaccination have the highest impact on the costs per HZ-case avoided in the DSA (Figure 4a). A decrease in costs of vaccination, e.g. by 50% per vaccine dose and to 5.20 Euros administration costs, lowers the ICER by 63%. In addition, varying the annual discount rate and annual HZ-recurrence influenced considerably the ICER (Figure 4a). The low PHN-related VE has a great impact on the costs per PHN-case avoided and increases the respective ICER by factor 4, which represents the general high impact of variations in PHN-related VE in health economic analyses (Figure 4b). Whereas costs of vaccination, annual discounting, and HZ-recurrence have the highest impact on ICERs related to PHN-case avoided and costs per QALY gained, HZ-incidence and treatment costs have only little impact on ICERs (Figure 4).

### Structural sensitivity analysis

**Figure 5:**
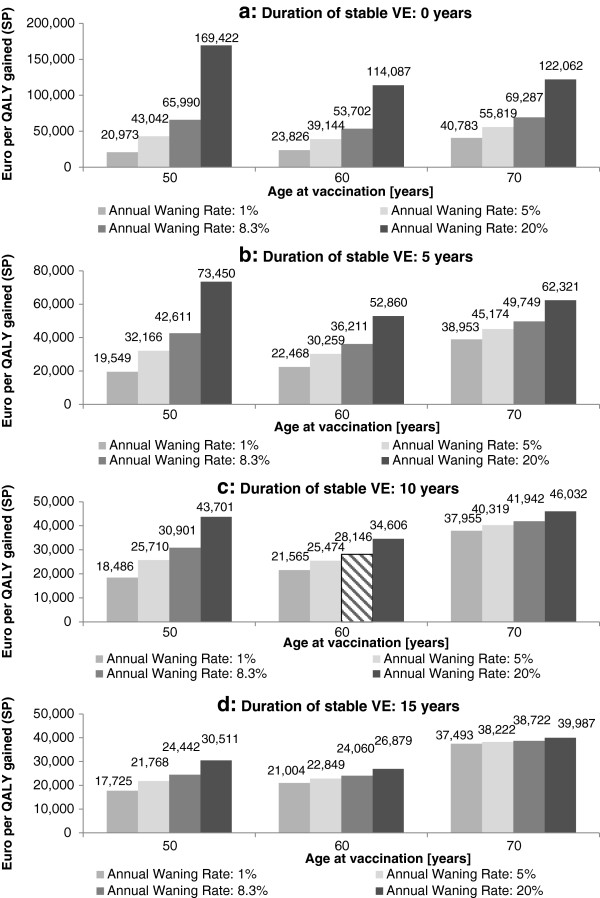
**Structural sensitivity analysis: impact of duration of stable vaccine efficacy (VE), annual waning of immunity and age at vaccination on cost per QALY gained.** QALY, quality adjusted life-year; SP, societal perspective. NOTE: Hatched bars represent base-case analyses.

In the best-case scenario cost-saving levels are almost reached. In contrast, the scenario least in favor for the vaccine accounts for very high ICERs (Table 4a). The variation of the period of stable VE following vaccination shows the logical negative correlation between duration of stable VE and ICERs. The lower the duration of stable VE following vaccination, the higher the ICERs are. ICERs decrease by factor 2.5 when increasing the duration from 0 to 20 years (Table 4b). Considering a variety of duration of vaccine induced protection and waning immunity rates, the analysis suggests that age 60 is most likely the optimal age at vaccination if the annual waning immunity rate is ≥5% (Figure 5). If the waning immunity rate is below 5%, age 50 seems to be the optimal age at vaccination. Furthermore, a high waning rate (≥8.3%) and a low duration of stable VE (<10 years) causes lower ICERs when vaccinating at age 70 when compared to vaccinating at age 50 (Figure 5a and 5b). With a higher duration of stable VE the impact of a varying waning immunity rate on ICERs by age at vaccination (Figure 5d).

The best case booster scenario is more cost-effective than the worst-case scenario independently from age at vaccination. However, the age-specific differences between both scenarios become smaller with increasing age: While ICERs at age 50 at vaccination differ between 30 to over 40% between both scenarios, the ICERs at age 80 at vaccination are almost equal (<0.1%, Table 4c). In Table 4d ICERs are shown by different duration of PHN. An increase from 6 to 9 month reduces ICERs by over 10%; an increase from 9 to 15 month causes a reduction by almost 20%. A PHN-duration of 3 years instead of 9 month decreases the ICER by almost 85% (Table 4d). Using age-specific baseline utility values causes an increase of about 30% compared to base-case figures. The ICER results in 36,629 Euros per QALY gained.

### Multivariate probabilistic sensitivity analyses

**Figure 6:**
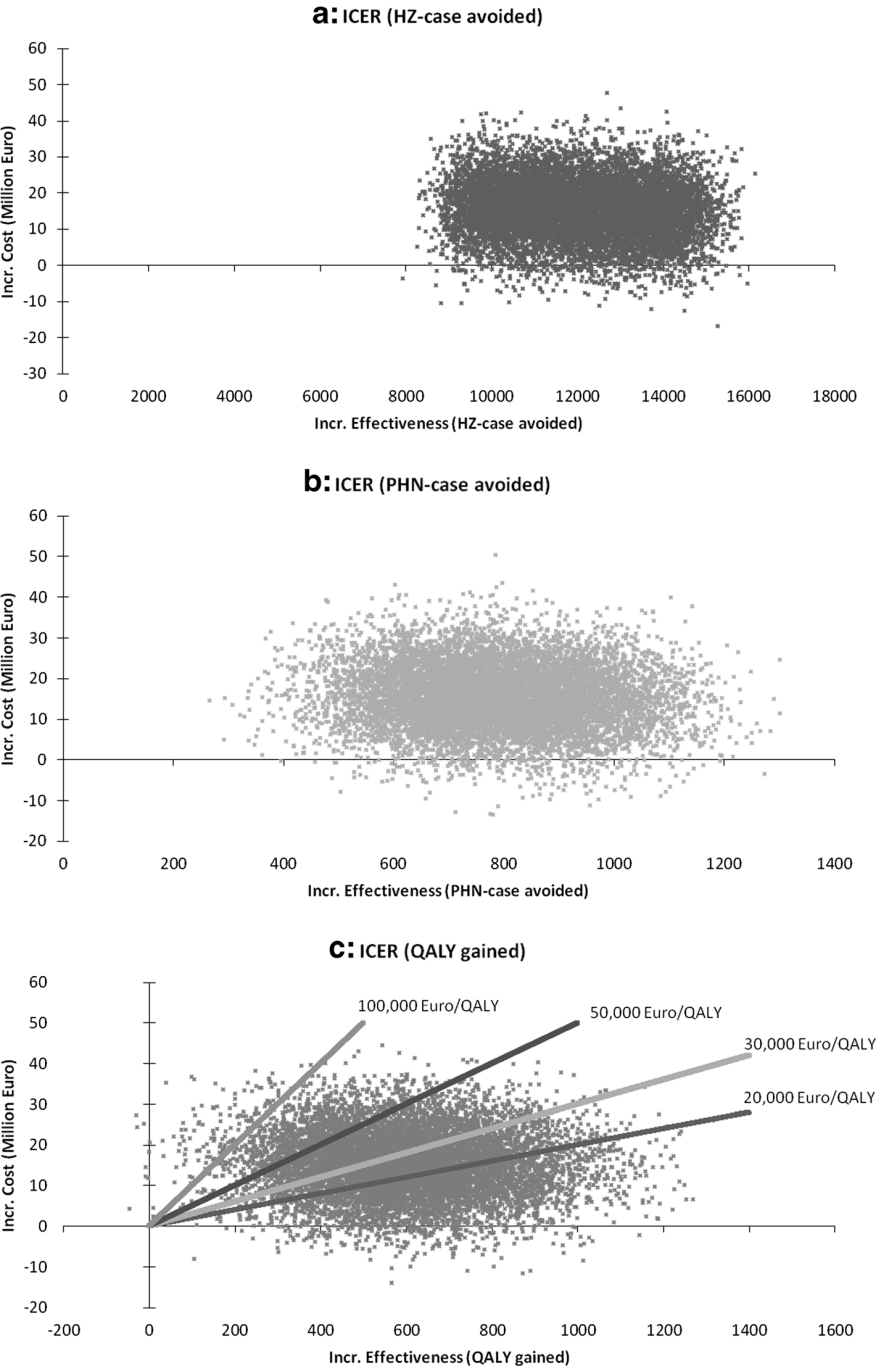
**Scatter plot: probabilistic sensitivity analysis by outcome.** ICER, incremental cost-effectiveness ratio; HZ, herpes zoster; PHN, postherpetic neuralgia; QALY, quality adjusted life-year; incr., incremental. NOTE: societal perspective; 10,000 Monte Carlo simulations.

The distributions of costs and respective outcome are presented in Figure 6. In the situation of HZ-cases avoided the scatter plot is compact (Figure 6a). The median ICER of 1,277 (95% CI 1,260 - 1,296) €/HZ-case avoided is about 10% lower than the respective base-case ICER. Approximately 3% of results are under the x-axis, representing cost-saving figures. In terms of costs per PHN-case avoided the results are distributed more widely with 1% in the cost-saving area. Compared to the PHN-related base-case the ICER of PSA is about 6% lower and resulted in 19,625 (95% CI 19,350 – 19,889) €/PHN-case avoided (Figure 6b). The scope of variation in cost per QALY gained reaches from cost-saving (2.5%) to less effective (<1%). Considering the costs per QALY gained, the calculated median ICER of 25,831 (95% CI 25,369 – 26,210) is about 8% lower than the ICER in base-case analysis (Figure 6c). The threshold 20,000 (30,000) Euro per QALY gained indicates that about 36% (60%) of all PSA results in Figure 6c are below the threshold. The higher WTP of 50,000 and 100,000 Euros/QALY gained, respectively, increases the proportion (87% and 99%) of PSA results located below the fictive thresholds (Figure 6c). When considering means instead of median values, the deviations between the respective PSA ICERs and the base-case ICERs are lower. Since the PSA ICERs are lower, our base-case assumptions as well as the base-case results are rather conservative.

## Discussion

We developed a static Markov-model and conducted various SA to assess the cost-effectiveness of routine HZ-vaccination in Germany and to identify the most cost-effective strategy when targeting specific age-groups for vaccination. HZ-vaccination of 20% individuals of a cohort of 1 million 50 year old persons at age 60 would avoid 20,000 HZ-cases and reduce the overall HZ-treatment costs by 10 million Euros. However, with a BCR of 0.34 for the base-case, our results show that vaccination against HZ and PHN is not a cost-saving measure. Univariate analyses revealed that the price had the greatest impact on ICERs in the DSA. In the break-even analysis with all other variable set at base-case scenario, a price per vaccine dose of <26.50 Euros caused cost-savings.

Varying several variables in favor for the vaccine (including a 50% reduction in the price per vaccine dose to 70 Euros) the intervention becomes almost cost-saving. However, under least favorable conditions (low estimates for HZ/PHN-related incidence and treatment costs, low HZ-recurrence rate, high utilities, low VE, a high waning immunity rate, high vaccine administration costs, and a high vaccine dose price) the model accounts very high ICERs. In general, health economic evaluations of new vaccines are often impacted by two important input factors: incidence of the target disease and VE. However, since the incidence data are rather reliable and range within narrow CIs, the wide ranges of VE (especially for the protection against PHN), which are based on trial data, mainly force the differences of these two extreme scenarios. Furthermore, the variation of the vaccine price (-50% vs. +50%) and the variation of disease recurrence seem to influence the ICERs, too. However, these two extreme scenarios of varying several input parameter either in favor for the vaccine or under least favorable conditions, can be seen as unlikely outer boundaries of the whole SA’s scope.

Due to decreasing VE by age, an increasing age at vaccination usually leads to higher ICERs indicating a lower cost-effectiveness. However, before the age of 60 the ICERs are not necessarily more favorable, due to lower HZ and PHN-related incidence and assumed waning immunity. Hence, targeting persons aged 60 years is likely the most cost-effective vaccination strategy. The variation in the annual waning rate of vaccine-induced immunity had rather limited impact on the ICERs. Altering the period of stable VE following vaccination from 0 to 20 years produces a relative wide array of ICERs. The combined analysis of varying waning immunity rates and durations of stable VE has a rather high impact on ICERs by age at vaccination. However, this analysis confirms that age 60 seems to be the optimal age at vaccination if the annual waning rate is ≥5%. The booster scenarios showed an increasing similarity between best and worst booster scenario with increasing age at vaccination. This confirms the assumption that independently from how a fictitious booster scenario is designed age of vaccination is one of the most relevant factors. The most cost-effective age of vaccination changes from 50 to 60, when moving from best to worst booster scenario. A variation of PHN-duration (6–15 month) had a moderate impact in ICERs. Whereas an extreme extension of PHN-duration up to 3 years downsizes the costs per QALY gained enormously. However, since recent studies confirm an average PHN-duration of several month, our base-case assumption of 9 month seems to be justified [29,65,66].

In terms of internal validity, we compared the epidemiology reported in the literature for Germany (used as model input parameter) and the model results. The model slightly overestimated HZ and accounted less than one percent more HZ-cases per age-group than represented by input-data. Regarding PHN-cases, the model calculated 4% more PHN-cases in age-groups 50 to 60 years. In older age-groups the overestimation was less than 1%. When implementing vaccination into the model, the accounted cases were reduced according to the VE implemented into the model. Thus, the model’s internal validity can be considered as good.

To date there is no other health economic evaluation of the HZ-vaccine from Germany. However, we identified 14 studies from other European countries [17,34,40,54,67-71] and North-America [56,72-75]. We found a wide range in vaccine prices from 43.85 to 438.5 PPP-Euros in one US study [72] and 81 to 147.32 PPP-Euros in the other studies. From SP for vaccination of individuals of 60 years results ranged from cost-savings [72] to 130,097 PPP-Euros per QALY gained [73]. From PP ICERs ranged from 6,809 [34], 1,200–46,968 [54] to 40,050 PPP-Euros per QALY gained [68]. Our base-case results range well within these international findings. However country-specific issues like vaccine-price, disease epidemiology, price levels, and treatment pathways as well as model-specific issues like model structure, specific assumptions, and dealing with uncertainty hinder a full comparability. Hence, more uniform methods in studies are needed to make results more comparable [76].

One limitation of our health economic model is the absence of utility-data considering the impact on health related QoL caused by HZ and PHN specific for Germany. Instead, we used data derived from a Canadian study without country-specific adaptation, which might not necessarily represent the real impact of HZ/PHN on QoL in Germany [14]. Other studies have reported a higher limitation on QoL caused by PHN [13,77]. Thus, the utilized values in our model might underestimate the impact on QoL due to PHN, whereas the HZ utilities used in our model might overestimate the impact of HZ on QoL [29,35,78]. However, we reduced this uncertainty by varying respective utility values within SAs. Furthermore, we set the baseline utility value for healthy individuals in base-case to 1. Thus, the impact of HZ and PHN and therefore the effect of the vaccine might be overestimated, since age-specific utilities among elderly tend to be lower than 1. Even though for health economic evaluation the utilization of accurate utility data for QoL assessments is critical, in Germany age-specific utility data for healthy but also for the most indications are scarce since cost-utility analyses do not have the relevance in decision-making in Germany as in other countries. We identified in a literature search in total five studies evaluating the QoL among healthy individuals in Germany [61,79-82]. Since all studies have certain limitations (e.g. only visual analog scale values was reported, no age-specific values reported, small study sample) we decided against using values of one of these studies for baseline utility values within the base-case analysis. However, by considering age-specific baseline utility values in SA we provided insights into this factor. The cost data used in the model were derived from a database from one large regional SHI in Germany and might not necessarily be representative for the whole population living in Germany. However, since countrywide treatment guidelines for HZ and PHN exist and since prices are mandatory for all SHIs in Germany, we believe that the utilization of these input-data is justified and representative. Furthermore our incidence data included both immune-competent and incompetent individuals. Since the live-attenuated vaccine is licensed for immune-competent individuals only, the incidence figures used in the model might be slightly overestimated. Our treatment cost input-data did not include privately covered expenditures for health-care services and over the counter drugs. Hence, costs from SP might be underestimated. However, since HZ and PHN-treatments are usually covered by the SHI, we suspect that the level of underestimation is low. As evidence was lacking concerning the duration and waning of vaccine-induced immunity, we had to make a few assumptions but included them in the SA. Finally, a real cost-effectiveness threshold concerning the WTP does not exist for the German health-care system. Therefore especially the ICERs and PSA results on costs per QALY gained have to be interpreted with caution. However, when comparing different age-groups to be targeted by routine HZ-vaccination and when comparing different scenarios (e.g. with and without booster vaccination), the lack of a cost-effectiveness threshold for Germany does not constitute a relevant limitation.

Our model provides valuable analyses and insights when considering the implementation of an efficient strategy for the prevention of HZ and PHN, and it will guide decision-making when developing a vaccination recommendation for Germany. First, this model reflects the efficacy of the vaccine quite well, since the HZ- and PHN-definitions as well as age-strata used in this model were similar to those used in the clinical-trials [21,29]. HZ-cases with a clinical diagnosis but also with a ‘suspected’ diagnosis were included in both data-sets. Second, the definition of PHN in the clinical trial was pain persisting more than 90 days after rash onset. This matches exactly with the definition in our input-data, in which HZ-cases became PHN-cases when they were diagnosed or received a PHN-specific medication at least three month after HZ-diagnosis. Second, a further strength of our analysis is the intensive parameterization during modeling. The base-case and the SAs demonstrate that on the one side a careful selection of reliable input-data is important, but on the other side a wide range of SAs has to be conducted to reduce uncertainty within model results. Especially HZ- and PHN-related incidence and VE have to be incorporated with caution, since these factors influence the analyses considerably. However, for our model incidence data were utilized from a large study recently conducted in Germany. These data correspond well with results from a nationwide incidence study in Germany and another retrospective data analysis in Germany, but also with study results from other countries [17,20,21,23,36,37,83-85]. Thus, this incidence input-data can be regarded as rather robust. SAs with variations of these data within the respective CIs have little impact on our results. However, vaccine characteristics are based on a less rich data fundament. While data on the VE in individuals from the age of 50 years is available, data on the duration of vaccine-induced protection and waning rates of vaccine-induced immunity is lacking and assumptions had to be made. Therefore, VE data were analyzed in SAs to assess the associated uncertainty. Based on one clinical-trial we assumed the period of stable VE following vaccination to be ten years. Since this assumption carries some uncertainty, we conducted a structural SA in which we varied the period of stable VE in order to assess the associated impact on results. Furthermore, evidence on the exact relative annual waning rate was utilized from literature. To analyze the impact of waning on ICERs we neglected the existence of a waning rate in one scenario and then subsequently increased it within SAs. A combined sensitivity analysis of varying waning immunity rates and durations of stable VE illuminated their combined impact on the optimal age at vaccination. Going one step beyond, we analyzed also the potential impact of differently designed fictitious booster scenarios on the results. This enables to assess booster scenarios way before evidence on the potential need for booster is established [86]. Finally, the SA in respect to HZ-recurrence shows a considerable impact of this parameter on the results. However neglecting HZ-recurrence has a lower impact than considering a high HZ-recurrence. This analysis shows that there is an urgent need to establish more evidence on HZ-recurrence on the long term view. Since HZ-recurrence but also HZ-booster vaccination are important parameters when conducting a health economic evaluation of HZ-vaccination in a given country, we believe that our results are also of high interest to other countries that consider the introduction of routine HZ-vaccination in their health-care systems. For our study we were able to use numerous country-specific input-data of high quality.

## Conclusion

Routine HZ-vaccination in the elderly is estimated to avoid a substantial number of HZ- and PHN-cases in Germany. However, under base-case assumptions the costs of vaccination are unlikely to be compensated by lower treatment costs. The vaccine price was identified to be crucial when considering overall cost and ICERs. In view of these findings, a responsible pricing seems needed. Targeting individuals aged 60 years is likely to be the most cost-effective vaccination strategy for the prevention of HZ and PHN in Germany. If another HZ-vaccine product becomes available on the market in the future, a comparative health-economic evaluation can be conducted by utilizing our model. However, results from head-to-head VE comparisons would be desirable for such an analysis.

## Competing interests

Potential conflicts of interest: Before initiation of this research, BU was an employee of Sanofi Pasteur MSD (provider of the Herpes zoster vaccine) from April 2008 to May 2010. For all other authors: No competing interests. The study was financially supported by the PhD funding program of the Robert Koch Institute.

## Authors’ contributions

BU initiated in the model design, developed the model, and drafted the manuscript. OW and FW participated in the model design, model validation and manuscript review. AS, TRe and GK participated in the model design and reviewed the manuscript. All authors validated the design and final results of the model. All authors read and approved the final version manuscript.

## Pre-publication history

The pre-publication history for this paper can be accessed here:

http://www.biomedcentral.com/1472-6963/13/359/prepub
